# Hypoxia-inducible factor (HIF) network: insights from mathematical models

**DOI:** 10.1186/1478-811X-11-42

**Published:** 2013-06-10

**Authors:** Miguel AS Cavadas, Lan K Nguyen, Alex Cheong

**Affiliations:** 1 Systems Biology Ireland, University College Dublin, Dublin 4, Ireland

**Keywords:** Hypoxia, HIF, FIH, PHD, Mathematical model

## Abstract

Oxygen is a crucial molecule for cellular function. When oxygen demand exceeds supply, the oxygen sensing pathway centred on the hypoxia inducible factor (HIF) is switched on and promotes adaptation to hypoxia by up-regulating genes involved in angiogenesis, erythropoiesis and glycolysis. The regulation of HIF is tightly modulated through intricate regulatory mechanisms. Notably, its protein stability is controlled by the oxygen sensing prolyl hydroxylase domain (PHD) enzymes and its transcriptional activity is controlled by the asparaginyl hydroxylase FIH (factor inhibiting HIF-1).

To probe the complexity of hypoxia-induced HIF signalling, efforts in mathematical modelling of the pathway have been underway for around a decade. In this paper, we review the existing mathematical models developed to describe and explain specific behaviours of the HIF pathway and how they have contributed new insights into our understanding of the network. Topics for modelling included the switch-like response to decreased oxygen gradient, the role of micro environmental factors, the regulation by FIH and the temporal dynamics of the HIF response. We will also discuss the technical aspects, extent and limitations of these models. Recently, HIF pathway has been implicated in other disease contexts such as hypoxic inflammation and cancer through crosstalking with pathways like NF*κ*B and mTOR. We will examine how future mathematical modelling and simulation of interlinked networks can aid in understanding HIF behaviour in complex pathophysiological situations. Ultimately this would allow the identification of new pharmacological targets in different disease settings.

## Introduction

Hypoxia is the cellular stress which occurs when oxygen demand exceeds supply. As a homeostatic response to this challenge, several classes of genes are up-regulated, which encode for proteins involved in angiogenesis, erythropoiesis and glycolysis, such as vascular endothelial growth factor, erythropoietin, most of the glycolytic enzymes and glucose transporters [1]. The possibility that higher organisms have direct oxygen sensing mechanisms, like those known for yeast and bacteria, has always interested biologists. A major advance in the knowledge of mammalian oxygen regulation came from studies on the hypoxic induction of erythropoietin (EPO). This glycoprotein regulates mammalian erythrocyte production and, as a result, oxygen delivery to tissues. EPO mRNA levels are several hundred folds induced in rodent liver and kidney in response to hypoxia and this induction was found to be effected by a nuclear factor induced by hypoxia, named hypoxia inducible factor (HIF) [2,3]. Later it was found that HIF is a dimer composed of an oxygen-regulated alpha subunit (HIF-1α, -2α and -3α) and a constitutively expressed nuclear beta subunit (HIF-1β, -2β and -3β) [4,5]. Although all the subunits are involved in generating the diversity of the transcriptional response to hypoxia [6], the core components are the 1α and 1β subunits.

**Figure 1:**
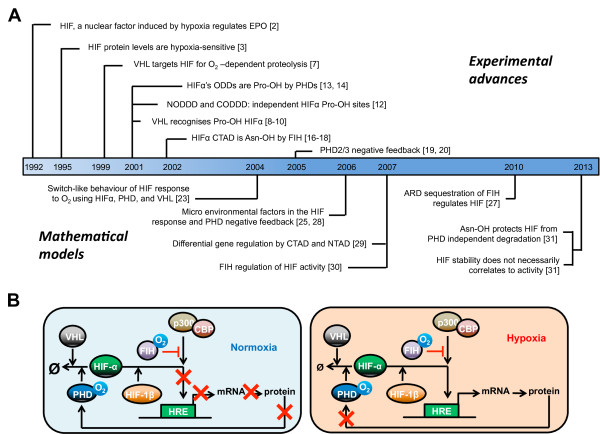
**Experimental and modelling advances in the HIF pathway.** (**A**) Historical profile of the major findings in the core components of the HIF pathway, alongside the major modelled behaviours. Numbers refer to the references. (**B**) Core elements of the HIF pathway: in normoxia, HIF-α is hydroxylated by PHD in presence of molecular oxygen (O_2_). This allows the binding of von Hippel–Lindau protein (VHL), eventually leading to HIF proteosomal degradation. HIF-α can also be hydroxylated by FIH, which interferes with the interaction with transcriptional coactivators p300 and CBP. In hypoxia, when demand of oxygen exceeds supply, the oxygen-sensitive PHD and FIH enzymes are inhibited. Thus HIF-α can accumulate, translocate into the nucleus and associate with HIF-β and p300/CBP, leading to formation of a transcriptional complex recognising hypoxia responsive elements (HRE) on the promoter of target genes. One such gene upregulated encodes for PHD, which creates a negative feedback loop.

In normoxia, the HIF-α protein levels are low due to constant ubiquitination-dependent degradation via the Von Hippel-Landau (VHL) E3 ligase protein [7], which recognises proline hydroxylated (Pro-OH) HIFα [8-10] on two independent sites: a carboxyl terminal and an amino terminal oxygen-dependent degradation domains (CODDD and NODDD, respectively) [11,12]. These hydroxylation reactions are catalysed by oxygen-sensitive prolyl hydroxylases (PHD) [13,14], establishing hydroxylation as a novel functional post-translational modification in signalling pathways [11]. Another level of control lies with the oxygen-sensitive asparaginyl hydroxylase FIH (factor inhibiting HIF), which hydroxylates the HIF alpha-protein and inhibits subsequent recruitment of the transcriptional co-activators p300 and CBP [15-18], thereby inhibiting the HIF transcriptional activity. In hypoxia, when less oxygen is available for PHD/FIH-mediated hydroxylation, HIF-α protein accumulates, translocates into the nucleus, associates with HIF-β and the co-activators p300/CBP to induce gene expression by binding to the conserved [A/G]CGTG hypoxia-responsive element (HRE) [1], including PHD2 [19] and PHD3 [20], establishing a negative feedback loop. A historical timeline of the major experimental findings together with the core components of the HIF network are shown in Figure 1. The ancient HIF response is conserved throughout the metazoans [21] and is an important physiological adaptation mechanism to conditions of low cellular oxygen, such as high altitude, ischemic stroke or myocardial infarction [1,11]. However, it can be hijacked in patho-physiological conditions such as cancer, where it allows for clonal selection of cancerous cells adapted to the hypoxic tumour microenvironment [1]. HIF-α is thus a promising anticancer drug target, and several compounds targeting HIF translation, degradation or transcriptional activity have already been approved [22].

In a linear biochemical cascade, effects of perturbations such as pharmacological inhibition of cascade components can be intuitively predicted and interpreted. However, cellular responses often appear to be mediated through highly interconnected and complex networks forming from multiple pathways crosstalk and feedback regulation. Such similar level of complexity featured by the hypoxia-induced HIF signalling network has motivated studies in which mathematical models are used to gain understanding of the emergent network behaviours. The focus of this review is to bring together the modelling efforts and discuss the models developed so far for the HIF signalling pathway. These models, implemented to run as computer programs, can generate simulations which provides a powerful tool to analyse and understand the HIF network behaviour in complex physiological situations, including inflammation and cancer. Moreover, model-based analysis would allow the identification of new pharmacological targets in different disease settings. We hope a comprehensive review of the available models of the HIF pathway will help lay a foundation for the development of more complete HIF models, as well as of future large-scale models integrating HIF and other pathways.

### Mathematical models of the HIF pathway

#### Overview of the HIF pathway models

Mathematical modelling of the HIF pathway was initiated by Kohn and colleagues [23], who also pioneered the development of molecular interaction map (MIM). Since then, a number of HIF models have been developed focusing largely on four topics which we will discuss in detail: (1) oxygen-mediated switch-like behaviour of HIF nuclear accumulation and transcriptional activity [23-27]; (2) HIF sensitivity to the molecular micro environment [25,28]; (3) role of FIH in the modulation of HIF activity and stability response [27,29-31] and (4) temporal dynamics of the HIF response to hypoxia [23,25-28,30,31]. A timeline of these models is given in Figure 1A.

**Figure 2:**
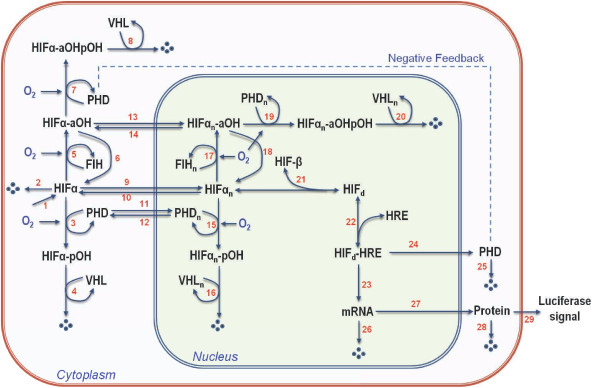
**Molecular interaction map (MIM) for the HIF network.** An example of the MIM used by our group for modelling the HIF response. The cellular localisation of the various moieties of HIF (free HIF, prolyl-hydroxylated pOH and asparaginyl-hydroxylated aOH), VHL, PHD and FIH are described to be either cytoplasmic or nuclear. Model reactions are numbered in red. Output of the MIM is the Gaussia luciferase signal under the control of HRE. Figure is reproduced from [31] with permission.

Modelling of a biochemical network typically begins with construction of a molecular interactions map which summarises the biological knowledge and provides a comprehensible and unambiguous graphical description of the network regulation. Figure 2 provides an example of such an interaction map for the HIF network [31]. The interactions are then formulated using precise mathematical terms which make up the mathematical model of the network. The available HIF models are primarily developed using ordinary differential equations (ODE) which are based on biochemical reaction kinetics, an approach appropriate for addressing network dynamic behaviours. Reaction kinetics described by either mass action or Michaelis-Menten laws are used to formulate the ODEs for each component of the network interaction map. In some models, model reduction using quasi-steady state analysis was performed for certain reactions, such as the hydroxylation reactions mediated by PHD and FIH and the interaction between VHL and prolyl-hydroxylated HIF protein. By using the Briggs-Haldane approximation [29], intermediary states can be assumed time invariant and neglected, thereby reducing the complexity of the system. Furthermore, values for the initial concentrations of each variable species and kinetic rate constants (e.g. association, dissociation, catalytics, synthesis, degradation) are either obtained from experimental measurements (in-house or from the literature) or estimated by numerically searching the parameter space for optimal fitting. A detailed summary of the more technical aspects of each of the developed mathematical models for the HIF pathway is presented in Table 1.

**Table 1:** Summary of the main features of HIF models

**Model reference**	**Modelled behaviour**	**Major assumptions**	**Findings/Predictions**	**Modelling approach**	**Parameter finding**
Kohn et al., 2004	HRE occupancy and/or mRNA expression in response to changes in oxygen concentration.	(1) Essential network behaviour (e.g. switch-like behaviour) encoded in core subsystem;	A switch-like behaviour for HIF is predicted to originate from	HIFα protein stabilisation and activity are modelled by numerically integrating a system of ODEs at each oxygen concentration.	k-space for optimised sharpness of HRE occupancy in response to oxygen concentration changes.
		(2) PHD confers oxygen dependence;	(i) rapid oxygen-independent HIFα synthesis		
		(3) Only one PHD isoform is dominant in each cell line;	(ii) oxygen-dependent HIFα degradation		
		(4) HIFα refers to either HIF-1α or HIF2α	(iii) a higher affinity of HIFα for PHD than HIF-1β		
		(5) Hypoxia responsive genes are activated by HIFα binding to HRE;	The negative feedback loop (HIF: PHD2) has non influence on this behaviour		
		(6) Hydroxylated moieties are equally likely to bind to and activate HRE;			
		(7) PHD promoter contains a HRE, which creates a negative feedback loop;			
		(8) HIFα is synthesized at a constant rate;			
		(9) HIF-1β concentration is constant.			
Kooner et al., 2006	HRE occupancy and/or mRNA expression in response to changes in oxygen concentration.	In comparison to Kohn’s model:	Oxygen-dependence of HIF-α: HIF-1β and HIF-α: HIF-1β: HRE complexes dissociation and HIF-α nuclear export is proposed to be the major mechanism responsible for the decrease in HIF protein and activity in the 0-0.5% oxygen tension.	HIFα protein stabilisation and activity are modelled by numerically integrating a system of ODEs at each oxygen concentration.	Experimental or assumption based mostly on NFκB model (e.g. mRNA and protein synthesis and degradation).
		(1) HIF dissociation from HIF-α: HIF-1β and HIF-α: HIF-1β: HRE complexes and nuclear export* is linearly dependent on oxygen;			
		(2) HIF-α and PHD associate in an oxygen-dependent way but the dissociation is oxygen-independent;			
		(3) Hydroxylation and ubiquination can occur both in the nucleus and cytosol;			
		(4) Negative feedback via PHD is not necessary.			
Yu et al., 2007	HRE occupancy and/or mRNA expression in response to changes in oxygen concentration	(1) Identification of the major pathways responsible for a behaviour can be analytically identified by extreme pathway analysis (EPA) instead of the more computationally-demanding numerical integration of a system of ODEs;	Switch-like behaviour is predicted to originate from the switching of a PHD-O_2_-VHL-dependent HIF degradation pathway in normoxia to an oxygen-independent degradation pathway in hypoxia.	Extreme pathway analysis for the analytical identification of key components responsible for the sharpness of the HIF response to oxygen, followed by analysis of fluxes through the pathway.	k-sets from Kohn et al. (2004).
		(2) Negative feedback via PHD is not included;			
		(3) HIF-α precursor species is a constant and therefore is integrated in the HIF synthesis rate constant.			
Qutub and Popel, 2006	Sensitivity of HIF protein to hydroxylation cofactors and PHD	Ascorbate is the key reducing agent responsible for keeping the Fe^2+^ pool in the cell, counteracting the oxidizing role of H_2_O_2_.	Two HIF responses to hypoxia are predicted according to the concentrations of PHD, Fe^2+^ and 2OG, which can reflect different cellular/environmental contexts. These 2 responses are either a steep switch-like response when all hydroxylation reactants are in excess, or a gradual increase, with a near-linear oxygen sensitivity when the reactants are limiting.	HIF protein levels are modelled by numerically integrating a system of ODE at each O_2_ level with different concentrations of key cofactors of the PHD hydroxylation reaction	Experimental or estimated from the model followed sensitivity analysis.
Qutub and Popel, 2007	Temporal effect on HIF protein stabilisation by succinate inhibition and PHD negative feedback.	This model builds on the one from Qutub and Popel (2006) and includes the effect of succinate, a product of the PHD hydroxylation reaction, on the ratio of PHD2 to HIF protein.	The ratio of PHD2 to HIF in different tissues and cell types is proposed to modulate the HIF accumulation to chronic hypoxia.	In addition to the approach in Qutub and Popel (2006), the model integrates the time course for PHD2 synthesised in response to hypoxia and the accumulation of succinate.	Experimental or estimated from the model followed sensitivity analysis.
			The model predicts a very sharp and transient accumulation of unhydroxylated HIF protein at high PHD2: HIF ratio, and a reduced but sustained HIF stabilisation at low PHD2: HIF ratios.		
			Accumulation of succinate under conditions of chronic hypoxia is predicted to inhibit the HIF hydroxylation reaction.		
Yucel and Kurnaz, 2007	Sensitivity of the angiogenic behaviour of a cancer cell to PHD and FIH.	(1) The angiogenic potential of a cancer cell is dependent on HIF-mediated VEGF expression which is differentially regulated by FIH and PHD;	Both PHD and FIH overexpression in hypoxia are predicted to decrease HIF-mediated transcriptional activation of VEGF. Only PHD is able to decrease the transcriptional activity to normoxic levels.	Concentrations of reaction species and kinetic reaction constants were inputted into GEPASI alongside with the governing equations following the principle of Mass action and Michealis-Menten kinetics.	Experimental or estimated from the model followed sensitivity analysis.
		(2) Compartmentalisation (nuclear, cytoplasmic and extracellular matrix) is included for HIF and VEGF, but not for the hydroxylases;			
		(3) FIH is a post-translational modificator of HIF, through Asn-hydroxylation, which sequesters HIF in a form that is unable to bind the co-activators p300/CBP after translocating into the nucleus.			
		(4) PHD is the regulator of HIF stability by promoting the proteasome-mediated degradation after the Pro-hydroxylation of HIF.			
Dayan et al. 2009	FIH controls a switch between C-TAD and N-TAD HIF target gene repertoires	(1) The C-TAD modified by FIH and the N-TAD modified by PHD function as independent RNA polymerase recruitment domains, controlling different subsets of genes;	N-TAD dominant genes are predicted to be induced during moderate hypoxia while C-TAD dominant genes are predicted to be induced in more severe hypoxia, when FIH loses its activity.	Standard ODE approach, HIF transcriptional activity is modelled as a gene induction function. This incorporates a parameter “q” where q=0 for FIH-independent and q>0 for FIH-dependent genes.	Numerical fitting of the parameters to experimental data was done through non-linear fitting with Mathematica.
		(2) No assumption of a switch-like behaviour;	The FIH sensitivity of a gene is proposed to be estimated from the ratio of mRNA fold change at 3% O_2_ and at anoxia.		
		(3) Only HIF-1α is modelled, and the data is fitted to a HIF-1α only cell line (LS174).	No switch-like behaviour is predicted: the model did not find a region in the parameter space (k-space) where two stable equilibriums could exist.		
Schmierer et al., 2010	ARD proteins sequestration of FIH modulates FIH activity	Building on the framework from Dayan et al. (2007), the module was extended at the conceptual level into taking into consideration:	The FIH/ARD interaction is predicted to provide a mechanism by which the hypoxic response threshold can be:	Standard ODE approach was used to modulate the HIF transcriptional activity and protein stability under different combination of hydroxylation status.	Numerical fitting of the parameters to experimental data was done through non-linear fitting with Mathematica.
		(1) Asn-OH-C-TAD-HIF can lead to a third subset of HIF target genes, which is only activated in moderate hypoxia;	(i) varied (range finding mechanism); (ii) ultrasensitive to oxygen level;	All simulation were done using the open source software XPP-AUT.	
		(2) ARD proteins can sequester FIH in an inactive state, until it is released at intermediate oxygen concentrations (moderate hypoxia)	(iii) sharpen the signal to response curves;	Steady state values calculated by running time course simulation at different oxygen levels until a steady state was achieved.	
		(3) HIF-1β, 2OG and Fe^2+^ are not limiting;	(iv) create a time delay for C-TAD hydroxylation upon reoxygenation (memory effect).		
		(4) Degradation of HIF-Pro-OH and HIF-α: HRE binding are fast compared to Pro-hydroxylation.			
Nguyen, Cavadas et al., 2013	Global temporal dynamics of the HIF: PHD: FIH network.	(1) The degradation of Pro-OH HIF via VHL binding is assumed to be an irreversible step;	The model predictions match in-house experimental data. The main findings are:	HIFα protein stabilisation and activity and protein level for PHD and FIH are modelled by numerically integrating a system of ODEs in different experimental conditions (such as oxygen concentration, PHD or PHD and FIH inhibition) over 12 hours period. This follows an iterative process of predictive hypothesis and experimental validation.	Numerical fitting of the parameters to in-house experimental data was done through non-linear fitting with Mathematica.
		(2) A generic PHD entity is considered for simplicity;	(i) a residual activity of FIH at low oxygen concentration which can be inhibited by siRNA;		
		(3) FIH is assumed to be mostly cytoplasmic;	(ii) HIF activity is not directly correlated to HIF protein expression levels;		
		(4) Hydroxylation and VHL-mediated degradation can occur in both nucleus and cytoplasm with similar kinetics;	(iii) silencing FIH under conditions of PHD inhibition increases HIF activity but paradoxically reduces HIF stability, explained by a role for FIH in controlling HIF protein stability.		
		(5) Asn-OH-HIF can be hydroxylated by PHD but Pro-OH-HIF cannot be hydroxylated by FIH due to the fast degradation kinetics of Pro-OH-HIF;			
		(6) Hydroxylation steps are mostly irreversible, although a small fraction could in theory be reversible;			
		(7) Both hydroxylated and non-hydroxylated HIF and PHD can shuttle between the cytoplasm and the nucleus;			
		(8) p300/CBP co-activators are not included and HIF-α/β dimer is assumed to be active;			
		(9) PHD is HIF-inducible, forming a negative feedback loop;			
		(10) Only nuclear HIF-α free of asparaginyl hydroxylation is assumed to be transcriptionally active.			

* HIF is exported to the cytoplasm to undergo degradation upon reoxygenation of hypoxic cells [34,35].

#### Switch-like behaviour in response to decreasing oxygen

**Figure 3:**
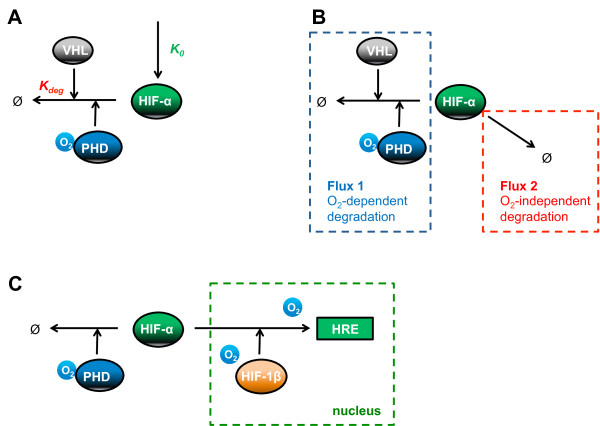
**Different model-based explanations for the switch-like behaviour.** (**A**) Hypoxia causes the oxygen-dependent HIF degradation rate (K_deg_) via PHD and VHL to be lower than the oxygen-independent HIF synthesis rate (K_0_) [23]. (**B**) Hypoxia causes the oxygen-dependent HIF degradation pathway (Flux 1) via PHD and VHL to be lower than the oxygen-independent pathway (Flux 2) [26]. (**C**) Oxygen regulates the activity of PHD as well as the HIF nuclear export and the dissociation rates for HIF: HRE and HIF-1α/1β complexes [33].

While the HIF-α response is experimentally reported to increase exponentially with reducing oxygen tension over the physiological range, there is instead a slight decrease in the HIF-α response at low oxygen levels (0.5% to 0% O_2_) [32]. This decrease is intriguing because the hydroxylation reaction by PHD requires oxygen as substrate and it is generally assumed that at levels close to anoxia, the HIF response is maximal. These observations gave rise to the hypothesis that HIF response could offer an on/off (a “switch”) mechanism for the onset of hypoxia-induced gene expression and that there is a sustained plateau of HIF response at very low oxygen tension [23,25-27,33]. Many of the available HIF models aim to theoretically explain this hypothetical switching mechanism, and at least three conceptually different explanations have been provided (Figure 3).

Starting from a comprehensive MIM of the HIF network, Kohn and colleagues reduce it to a core subsystem comprising of HIF-α, HIF-β, PHD2, PHD3, VHL and a generic HIF target gene containing a hypoxia responsive element [23]. Under normal oxygen conditions, the oxygen-independent synthesis rate of HIF-α is assumed to be lower than the oxygen-dependent degradation rate arising from a fast hydroxylation reaction by the PHD enzymes. Thus almost every HIF protein synthesised would be hydroxylated and degraded before being able to bind HIF-β. As the level of oxygen concentration decreases, PHD activity is increasingly reduced, resulting in decreasing HIF-α degradation rate which at a threshold of oxygen level, becomes lower than the HIF-α synthesis rate. Subsequently, accumulated HIF-α associates with HIF-β, leading to HRE occupancy. Kohn’s model manages to simulate a sharp increase in HIF response and a plateau phase at low oxygen tension. Interestingly, the model analysis proposes that this sigmoidal, switch-like behaviour occurs only if the affinity of HIF-α for PHD is assumed greater than for HIF-β [23]. The switch is further investigated by Yu and collaborators using a Boolean based extreme pathway analysis approach [26]. In Yu’s model, the HIF network is further simplified by excluding reactions which are shown not to be required for the switch-like behaviour, such as the PHD negative feedback loop [23]. In addition, Yu’s model considers 3 major pathways for HIF degradation [26]. This modified model predicts that the switch-like behaviour is caused by switching from a PHD-O_2_-VHL-dependent HIF degradation pathway in normoxia to an oxygen-independent pathway in hypoxia. The latter may be due to the reported binding of p53 to HIF in anoxia (<0.2% O_2_) which leads to induction of HIF degradation [34].

Although the models by Kohn *et al* and Yu *et al* both describe a plateau in the HIF response at very low oxygen tension, this is however not in total agreement with the experimental data they have used, which shows instead an attenuation of the HIF response at oxygen levels close to anoxia [32]. In order to address this discrepancy, Kooner and colleagues have extended Kohn’s model by considering the role of oxygen in mediating the reactions of HIF-α with PHD, HIF-β and HRE, as well as its nuclear export [33]. Their model assumes that HIF-α and PHD associate in an oxygen-dependent manner but the dissociation reaction is oxygen-independent. Furthermore, HIF-α: HRE and HIF-1α: HIF-1β dissociations as well as HIF-α nuclear export are assumed to be linearly dependent on oxygen concentration. Using data on the export of HIF-α from the nucleus and its degradation in the cytoplasm following reoxygenation [35,36], Kooner’s model is able to reproduce the attenuation effect of the HIF response at oxygen concentrations below 0.5%. This suggests that the localisation of HIF and other core proteins can be significantly important for the modulation of the HIF response.

#### Importance of the molecular microenvironment

**Figure 4:**
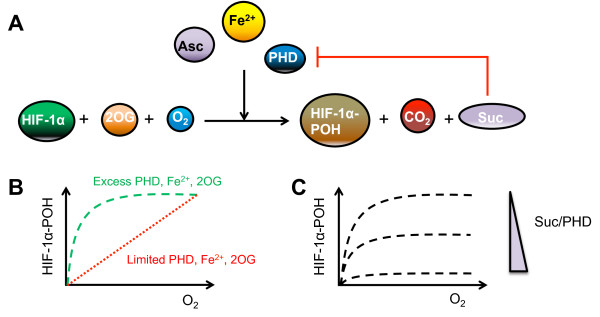
**Different model-based explanations for the effect of the PHD hydroxylation reaction on the HIF response.** (**A**) The PHD hydroxylation of HIF-1α protein requires molecular oxygen (O_2_), iron (Fe^2+^), 2-oxoglutarate (2OG) and ascorbate (Asc) as reactants, producing succinate (Suc) and carbon dioxide (CO_2_). (**B**) In the presence of abundant PHD and hydroxylation cofactors, there is a step decrease in prolyl-hydroxylated HIF (HIF-1α-POH) with decreasing oxygen. However, this decrease is linear under limited PHD2 or cofactors [25]. (**C**) Increasing the ratio of succinate to PHD leads to increased succinate inhibition of PHD from negative feedback (**A**), resulting in decreased prolyl-hydroxylated HIF [28].

In addition to oxygen, the hydroxylation reaction catalysed by PHD requires both Fe^2+^ and 2-oxoglutarate (2OG, also known as α-ketoglutarate) as reactants [11]. The reaction can also be affected by other micro environmental factors, including ascorbate (vitamin C). Their levels vary across tissues, which may affect the tissue-specific HIF response via the regulation of PHD activity [37]. Furthermore, 2OG is converted to succinate (Figure 4A), both of which are part of the Kreb’s cycle, and directly link PHD activity to cell metabolism [38,39].

To analyse the effect of the molecular environment on the HIF response, Qutub and Popel constructed a model of the HIF network incorporating the levels of Fe^2+^, ascorbate and 2OG [25]. When the cellular levels of PHD, 2OG and Fe^2+^ are in excess, their model predicts a steep drop in HIF hydroxylation with decreased oxygen. However, if any one of the reactants is limiting, a near-linear response to oxygen is observed instead. Moreover, when two or more reactants are limiting, HIF-α hydroxylation is greatly reduced with a significant decrease in sensitivity to oxygen (Figure 4B). The effect of ascorbate is found to be more complex, and an intermediate response is instead predicted when ascorbate level is limiting [25]. These predictions open up potential therapeutic strategies for modulating HIF-α hydroxylation by varying the metabolic environment of cells. A later model [28] includes the level of succinate, which has been shown to negatively feedback onto the hydroxylation reaction [38,40-42]. The prediction from this model is that a high succinate to PHD ratio at the beginning will result in a decrease in HIF-α hydroxylation and increase in HIF activity (Figure 4C). This is relevant to the condition when succinate level is higher than normal, such as in the case of succinate dehydrogenase deficiency [41,42].

#### Role of FIH in shaping the HIF response

**Figure 5:**
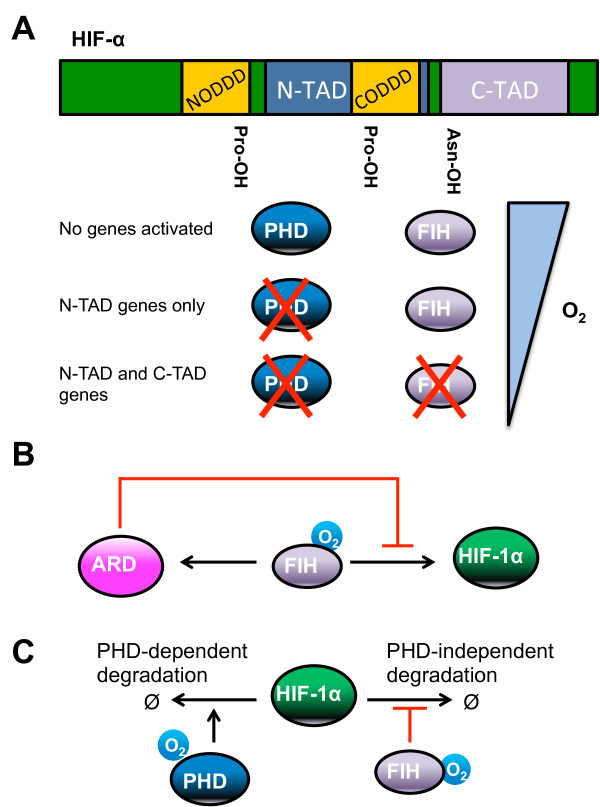
**New roles for FIH in the regulation of the HIF response.** (**A**) The HIF-α protein contains two independent transcriptional activation domain (N-TAD and C-TAD), the N-TAD overlaps with the CODDD. PHD enzymes hydroxylate the prolyl residue present in the N-TAD, while FIH hydroxylates the asparaginyl residue in the C-TAD. In high oxygen concentration, both PHD and FIH are active, resulting in no HIF-regulated genes activated. As the oxygen tension decreases, PHD is inactivated, resulting in expression of N-TAD-sensitive genes. In strong hypoxia, both PHD and FIH are inactivated, resulting in expression of N-TAD and C-TAD-sensitive genes [29]. (**B**) FIH can hydroxylate either ARD or HIF-α proteins. Sequestration of FIH by ARD inhibits HIF asparaginyl hydroxylation [27]. (**C**) HIF-α can be degraded via either PHD-dependent or -independent pathways. FIH hydroxylation of HIF is proposed to protect HIF degradation via the PHD-independent pathway [31].

The HIF-α protein contains two independent transcriptional activation domains (N-TAD and C-TAD [43-46]). PHD enzymes hydroxylates HIF-α at prolyl residues present in the N-TAD, while FIH hydroxylates at the asparaginyl residue in the C-TAD (Figure 5A). This is mathematically represented in a model by Dayan and colleagues, which aims to simulate the sensitivity of a set of genes to FIH activity over an oxygen gradient [29] (Figure 5A). Some genes are proposed to be dependent on the N-TAD only, while others require an additional intact non-asparaginylated C-TAD. Given that PHD has a higher affinity for oxygen than FIH [47], the differential and spatially dependent gene upregulation by HIF is attributed to the sensitivity of certain genes to an oxygen gradient which inactivates PHD but not FIH (mild hypoxia; N-TAD-sensitive/C-TAD-insensitive genes) or both PHD and FIH (strong hypoxia; N-TAD/C-TAD-sensitive genes) [29,46] (Figure 5A). This model prediction appears to agree with experimental data on FIH-sensitive and insensitive genes [46]. However, a group of other genes (including BNIP3 - BCL2/adenovirus E1B 19 kD interacting protein 3) does not fit the model, and FIH overexpression instead increases their expression. For this observation, Dayan and colleagues propose the existence of an unknown C-TAD-dependent repressor which is activated and de-represses this particular group of genes. Schmierer and colleagues suggest an alternative explanation based on their model, which predicts that a group of genes (such as BNIP3) is actually activated by an Asn-hydroxylated-C-TAD HIF-α, and thus not dependent on CBP/p300 [27]. Schmierer’s model also includes the hydroxylation of other ARD (ankyrin repeat domains) proteins by FIH and assumes that there is competition between ARD proteins and HIF for FIH (Figure 5B). Thus, as oxygen tension decreases, the model predicts that FIH catalytic activity decreases and is less likely to hydroxylate HIF at its C-TAD [27]. Furthermore, Schmierer’s model of ARD/HIF/FIH interactions is predicted to generate a time delay for C-TAD hydroxylation and encode a memory effect of the hypoxic episode [27]. The more severe the hypoxic exposure, the longer will it take for FIH to be free from ARD sequestration upon re-oxygenation, resulting in non-Asn-hydroxylated C-TAD-HIF moiety lingering longer in the nucleus.

The model developed in our lab considers the HIF-α network as a dynamic system affected by both oxygen concentration and the duration of exposure to hypoxia [31]. The HIF-α moieties (free, prolyl- and asparaginyl-hydroxylated forms) are treated and described separately in the model, as are their cytoplasmic/nuclear localisation (MIM shown in Figure 2). This model incorporates both PHD and FIH as the major HIF hydroxylases and also accounts for the negative feedback regulation due to HIF-induced expression of PHD. Model calibration is carried out based on experimental data generated in-house which consist primarily of the time-course dynamic data up to 12h post hypoxic stimulation of HIF stabilisation and transcriptional activity using a pan-hydroxylase inhibitor (Dimethyloxalylglycine, DMOG) or a selective PHD-inhibitor (JNJ1935 [48]). The model is able to satisfactorily simulate the HIF-1α temporal response to different oxygen levels and also to increasing concentrations of DMOG and JNJ1935 (reflecting PHD+FIH or PHD inhibition). It is also able to simulate the modest effect of FIH silencing on HIF-1α activity observed *in vivo* in mice lacking FIH [49]. Moreover, the model predicts an unusual role for FIH in regulating the stability of HIF by protecting HIF-1α from non-PHD mediated degradation (Figure 5C). This prediction is further supported by quantitatively analysis of a reduced model containing core interaction module of the HIF network, and closely matches experimental data using an overexpressed HIF-1α with mutated prolyl residues (making it non-hydroxylable by PHD). Thus this model reassures the known wiring structure of the HIF network and more importantly a new role for FIH in regulating HIF stability is proposed from the model and validated experimentally.

#### Temporal dynamics of the HIF response to hypoxia

The transient accumulation of HIF-α during hypoxic time-course exposure is a well characterised feature of *in vitro* systems, described to be due to the presence of negative feedback loops [25,28,30,31,50]. The best described mechanism for this decrease is the up-regulation of HIF-regulated PHD 2 and 3 enzymes [19,20], although other HIF-regulated feedback such as mir-155 [50] can also affect the transient response. Qutub and Popel analyse the effect of varying the ratio of PHD: HIF synthesis and predict that this might be a mechanism to generate very different HIF responses, possibly to allow different cell types to respond differently to a hypoxia stimulus [28]. At higher PHD: HIF synthesis ratio, the HIF response is sharp and transient. When the ratio is lower, the response is delayed but does not attenuate, in close agreement to experimental data in HEK293 cells [51]. Their model assumes that the PHD: HIF synthesis ratio can be controlled by 3 feedback loops: autocrine HIF up-regulation, negative feedback through HIF up-regulation of PHD2 and succinate production inhibition [28].

## Discussion

### Usefulness of current models

While many of the molecular components of the HIF pathway have been identified and characterised, the dynamics of their interaction within the network are less well understood. Knowledge of the network components alone do not warrant a wholistic understanding of the system, particularly by simple intuition. The collection of models developed so far for the HIF pathway has provided different quantitative frameworks upon which emergent properties of the network as a whole can be obtained, which is crucially important in understanding effects to perturbation such as drug responses. Some of these models provide plausible mechanistic explanations for previously observed experimental observations which are nontrivial otherwise. For example, the molecular mechanisms leading to a HIF switch-like response to decreasing oxygen levels have been extensively modelled [23,26,33] based on the original experiments by Jiang and co-workers [32]. These *in silico* studies can help in the elucidation of the pathophysiological role of the oxygen gradient established in a tumour microenvironment. Furthermore, modelling and simulation can suggest new testable hypotheses which guide further experiments in a rational way. The model from Dayan and co-workers [29] proposed a new paradigm of hypoxic differential gene regulation within this tumour oxygen gradient. Given the differences in oxygen sensitivity for PHD and FIH, a sequential inhibition of PHD followed by FIH is predicted down the oxygen gradient. Thus hypoxia is proposed to induce specific HIF-regulated genes in a spatially controlled way depending on their C-TAD or N-TAD sensitivities [29]. It is normally assumed that HIF protein stabilisation is indicative of HIF transcriptional activity. However, work from our combined experimental and modelling study shows that this is not necessarily true, and HIF activity is tightly controlled by the activity of the asparaginyl hydroxylase FIH [31]. Furthermore, our mathematical model predicts that FIH, through asparaginyl-hydroxylation, can protect HIF from PHD-independent degradation mechanisms (Figure 5C), establishing a new role for FIH in HIF stabilisation.

### Limitations of the current models

The models developed so far have focused on understanding the relationship among oxygen levels, a limited number of micro environmental factors and the HIF response (protein stability and transcriptional activity) using the canonical components of the HIF pathway (HIF, VHL, PHD, FIH and the HRE element). While the main components can broadly describe the HIF response, several assumptions have been made while constructing the models. Sometimes these assumptions were taken to simplify the modelling process but more often than not the assumptions are due to lack of experimental data. Indeed, most of the HIF models to date have been constructed based on published third party data although some models including ours [29,31] have benefited from a process of iterative experimental and mathematical validations. Moreover, the limitation in the availability of dynamic experimental data has restricted most models to focus on steady-state behaviour, except the more recent ones [31].

A common assumption has been that the PHD isoforms 1, 2 and 3 behave as a single entity, i.e. they respond similarly to oxygen levels, are responsible for maintaining the HIF baseline normoxic levels and are hypoxia-inducible to provide a negative feedback loop. Although the oxygen sensitivities of the PHD enzymes are generally similar [47], the PHD2 isoform is, for many cell lines, the most abundant and considered to be the main prolyl-hydroxylase for HIF stabilisation in normoxia [52]. Experimentally however, the other two isoforms are not redundant, as PHD1 and PHD3 have been shown to contribute to the HIF regulation in certain cell lines [53]. Furthermore, compartmentalisation of the proteins involved in the HIF response was rarely considered in most models, even though PHDs were shown to have specific localisation in the cell which could affect their activities [54]. Similarly, HIFα exists as three different subunits (1, 2 and 3) and have been shown to regulate specific sets of genes [55,56]. While it is technically possible to differentiate among the subunits through isoforms specific gene silencing, most mathematical models simply assume that hypoxia response is mediated by HIF-1α only. Our group has bypassed this challenge by modelling the hypoxia response in HEK293 cells lacking the HIF-2α subunit [31].

The lack of signalling crosstalks in the current models reflects the technical challenges to obtain high quality data on the dynamics of different transcriptional factor activities (such as for HIF with NF*κ*B) and to measure the abundance/modification states of signalling proteins. Fortunately, there have been recent advances which have facilitated the generation of quantitative experimental data suitable for mathematical modelling. For example, the simultaneous transfer of proteins from multiple gel-strips onto the same membrane (multi-strip western blot) has increased data output per single blotting cycle and allowed simultaneous monitoring of proteins while reducing signal errors [57,58]. Also, our group has initiated the use of secreted luciferases as a non-invasive method for monitoring the temporal dynamics of transcriptional activity [31,59,60].

### Opportunities for future modelling work

**Figure 6:**
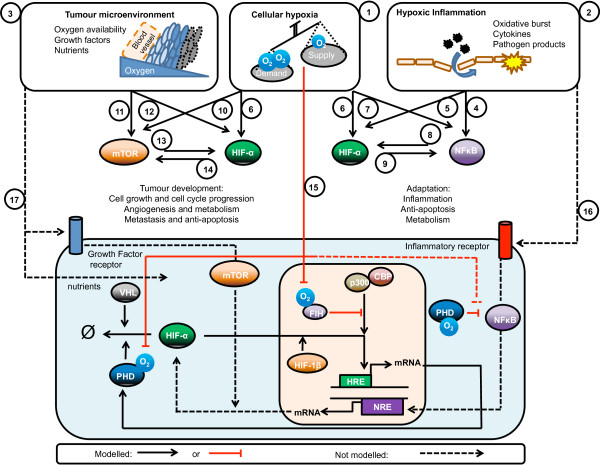
**Opportunities for further modelling work: HIF crosstalk to mTOR and NFκB in cancer and inflammation.** Hypoxia, the cellular condition when oxygen demand exceeds oxygen supply (1) is present in several physiological and pathophysiological processes including inflammation (2) were hypoxia is induced as a result of the highly metabolically active inflammatory cells and reduced blood supply associated with a disrupted vasculature; and cancer (3) were the highly proliferative cancer cells can be very far away from the vasculature. NFκB is classically activated by inflammatory stimulus (4) and has recently been appreciated to be regulated by hypoxia (5), both of these stimulus are present in regions of chronic inflammation and can also activate HIF (6,7). Furthermore, these two transcription factors show a significant degree of crosstalk with NFκB transcriptionally regulating HIF (8) and HIF regulating NFκB activity (9). mTOR is affected by hypoxia at multiple levels (10) and is activated in cancer (11). HIF is overexpressed in cancer, due to both tumour hypoxia (6) and mutations in tumour suppressor genes (12). Importantly, mTOR transcriptionally regulates HIF in response to growth factors (13) and HIF regulates for growth factor receptors and adaptor proteins which can affect mTOR signalling (14). While most of the mechanisms of the effect of cellular hypoxia on the HIF response have been modelled (15, continuous lines), the HIF/hypoxia crosstalk to NFκB and mTOR and the outcome of the interaction of these pathways in inflammation and tumour development are still open opportunities for further modelling research (16,17, dashed lines).

Overall, most of the modelling effort up to date has been centred on the *in vitro* HIF response to hypoxia with the HIF pathway being the sole focus (Figure 6). However, HIF is known to have important roles in normoxia, and more complex physiological conditions governed by extensive crosstalk to other pathways. Interestingly, but perhaps not surprisingly due to the increased complexity of the system, mathematical modelling of the crosstalk and synergism with other pathways such as NFκB [60,61] and mTOR [39,62,63] have not been considered. We will focus on NF*κ*B and mTOR as examples of topics for further research due to their significant degree of crosstalk during two pathophysiological conditions of both basic and pharmacological interests: inflammation and cancer.

### HIF and NF*κ*B crosstalk during hypoxic inflammation

Sites of chronic inflammation including arthritic joints and inflamed intestinal mucosa demonstrate decreased oxygen availability [61,64,65]. This is likely caused by increased oxygen demand from the inflamed tissue [66] as well as from the vasculopathy that leads to reduced blood perfusion [67,68]. In this context both HIF and NF*κ*B seem to play important functions. HIF can have both pro-inflammatory and anti-inflammatory roles, dependent on the cell where it is expressed, while NF*κ*B works primarily as regulator of inflammatory and anti-apoptotic gene expression [69]. In this hypoxic inflammation context, HIF and NF*κ*B seem to share sensitivity to similar stimulus. HIF is affected by reactive oxygen species ROS from the oxidative burst [70] and can be independently activated by hypoxia at the protein level and by bacterial lipopolysaccharide, tumour necrosis factor *α*, and interleukin-18 [70-74] in a mechanism involving the NF*κ*B pathway. Furthermore, NF*κ*B has also been reported to play a role in hypoxia-induced HIF-1*α* mRNA expression [71] and in keeping basal levels of HIF-1*α* gene expression [72,75]. Converserly, NF*κ*B has also been shown to be regulated by hypoxia and hypoxic mimetics [76,77], several components of the NF*κ*B pathway are hydroxylated by prolyl and asaparaginyl hydroxylases [78], and there is also compelling evidence for a role of HIF in the regulation of NFκ signalling [69,79,80], these two transcription factors seem to be able to integrate similar stimulus and to have an extensive crosstalk in the regulation of several inflammatory genes including cyclooxygenase 2 [60,81] and IL-1β [82]. Further understanding of this crosstalk with the help of mathematical modelling can provide a better understanding of gene regulation in hypoxic inflammation (Figure 6).

### HIF and mTOR crosstalk in cancer

In the complex process of cancer development, cells need to accumulate mutations that allow them to escape the intrinsic cellular and extrinsic environmental constraints on proliferation [83]. Solid tumors, where the process of tumor expansion exceeds the development of blood vessels, associated with the fact that the new blood vessels are aberrant and have poor blood flow [84], results in a hypoxic tumour microenvironment. Processes regulated by hypoxia in cancer extend from angiogenesis, glycolysis and growth-factor signalling to immortalisation, genetic instability, tissue invasion, metastasis, apoptosis and pH regulation. Most of the hypoxia-induced pathways promote tumour growth, but apoptosis is also induced by hypoxia. HIF-1α and HIF-2α protein is overexpressed in several primary tumours and this is associated with increased patient mortality, indicating that the HIF pathway promotes oncogenesis and/or cancer progression [1]. The balance of these pathways might be critical for the effects of hypoxia on tumour growth [1,84]. The mammalian target of rapamycin (mTOR) is a highly conserved kinase which can integrates signals from nutrients (amino acids and energy) and growth factors (in higher eukaryotes) to regulate cell growth and cell progression co-ordinately [85]. Its classical targets being the ribosomal p70S6 kinase (p70S6K) and eIF4E-binding protein, which lead to enhancement of translation and transcription, enabling cell growth and cell cycle progression from G1 to S phase. Pathways upstream of mTOR and mTOR themselves are activated in cancer. Insulin, angiotensin-II and epidermal growth factor have been shown to up-regulate HIF in the presence of molecular oxygen [62,86,87] and mTOR inhibition decreases tumour progression partially to decreased neo-vascularisation [88], indicating mTOR as a regulator of HIF by increasing its mRNA translation. Conversely mTOR signalling can also be affected by HIF and hypoxia, HIF target genes involved in cell proliferation and viability (insulin-like growth factor receptor-2 and insulin-like growth factor receptor binding protein-1, [86]) can further amplify mTOR signalling, and hypoxia can directly impact on mTOR signalling at multiple points [63], in a mechanism where the crosstalk between two pathways can potentiate cancer development (Figure 6). Mathematical modelling of these crosstalks is expected to provide important hints on the key therapeutic target nodes that could disrupt cell proliferation.

## Conclusions

Most of the modelling studies on the HIF pathway have been concerned with the core components of the pathway: HIF, PHD, FIH and VHL, which seem to suffice in providing the system with the characteristic network features: switch-like behaviour and attenuation under prolonged hypoxia. This supports the view that essential behaviour of biological systems is often encoded within small “core” subsystems, while additional complexity may serve to fine tune this behaviour. The additional complexity can be encoded by changes in the molecular microenvironment [25,28,38,40-42] and brought about by feedback loops [25,28,30,31,50].

While all models are supposedly wrong, some are useful [89]. This apparent limitation, though, has a positive implication: failure of a model to reproduce experimental data often indicate the presence of additional elements and prompt further investigations into the regulatory mechanisms of that same system. Mathematical modelling is therefore a valuable tool in aiding experimental biologists to better understand the HIF pathway by providing explanations for complex and sometimes counter-intuitive behaviours and offering predictions on missing protein partners or unknown functional relationships between components. Furthermore, modelling of the HIF response in more complex biological settings such as cancer and inflammation, where HIF is known to interact with major signalling pathways activated in these contexts, will facilitate biologists in formulating new hypothesis to explain complex behaviours.

## Abbreviations

2OG: 2-oxoglutarate (α-ketoglutarate); ARD: Ankyrin repeat domain; Asn: Asparagine; BNIP3: BCL2/adenovirus E1B 19 kD interacting protein 3; CBP: CREB-binding protein; C-TAD: Carboxyl-terminal activation domain; CODDD: Carboxyl-terminal oxygen dependent degradation domain; DMOG: Dimethyloxalylglycine; HIF: Hypoxia inducible factor; HRE: Hypoxia-responsive element; JNJ1935: JNJ-42041935; MAPK: Mitogen activated protein kinase; MIM: Molecular interactions map; mTOR: Mammalian Target of rapamycin; NFκB: Nuclear factor kappa B; N-TAD: Amino-terminal activation domain; NODDD: Amino-terminal oxygen dependent degradation domain; ODE: Ordinary differential equations; OH: Hydroxyl; PHD: Prolyl Hydroxylase Domain proteins; PI3K: Phosphatidylinositol-3-kinase; Pro: Proline; VHL: Von Hippel–Lindau protein.

## Competing interests

The authors declare that they have no competing interests.

## Authors’ contributions

MASC, LKN and AC wrote the paper. All authors read and approved the final manuscript.
